# A Case of Polymerase Proofreading‐Associated Polyposis: Challenges in Genetic Diagnosis

**DOI:** 10.1002/jgh3.70240

**Published:** 2025-07-29

**Authors:** Haruka Ito, Akiko Chino, Arisa Ueki, Keika Kaneko, Shoichi Saito

**Affiliations:** ^1^ Department of Gastroenterology Cancer Institute Hospital of Japanese Foundation for Cancer Research Tokyo Japan; ^2^ Department of Clinical Genetics Cancer Institute Hospital of Japanese Foundation for Cancer Research Tokyo Japan

**Keywords:** colonic polyposis syndrome, hereditary syndrome, polymerase proofreading‐associated polyposis

## Abstract

**Background:**

Polymerase proofreading‐associated polyposis (PPAP) is a rare autosomal dominant hereditary syndrome caused by germline pathogenic variants in the POLE or POLD1 genes. It is clinically similar to familial adenomatous polyposis (FAP) and Lynch syndrome, making diagnosis difficult. Although the number of reported cases is increasing globally, PPAP remains underrecognized, particularly in Japan. Accurate diagnosis often requires comprehensive genetic testing, including multi‐gene panel analysis and variant reinterpretation.

**Case Presentation:**

We report a rare case of PPAP in a 50‐year‐old woman with a complex clinical history involving multiple primary malignancies. The patient developed ovarian cancer in her 20s, followed by endometrial and contralateral ovarian cancers in her 30s. She was also diagnosed with early‐stage colorectal cancer and polyposis, for which she underwent total colectomy with ileorectal anastomosis. Initially, she was suspected to have FAP or Lynch syndrome, but genetic testing revealed no pathogenic variants in APC, MUTYH, or mismatch repair genes. Subsequent multi‐gene panel testing identified a POLE variant of uncertain significance (VUS), which was later reclassified as likely pathogenic. Based on this reinterpretation and her clinical phenotype, a diagnosis of PPAP was made. Her disease course included recurrent rectal polyps and carcinoma after colectomy, as well as breast cancer. No upper gastrointestinal polyposis was observed.

**Conclusion:**

This case represents one of the few reported instances of PPAP in Japan and illustrates the diagnostic complexity of hereditary polyposis syndromes. It highlights the critical role of multi‐gene panel testing and the importance of variant reinterpretation in establishing a definitive diagnosis. Continued surveillance and multidisciplinary care are essential for managing patients with PPAP.

## 1 Introduction

Polymerase proofreading‐associated polyposis (PPAP) is a rare autosomal dominant hereditary syndrome caused by germline pathogenic variants in the POLE or POLD1 genes. These genes encode DNA polymerases responsible for proofreading and correcting errors during DNA replication, and their dysfunction leads to an increased risk of colorectal adenomas and various malignancies. PPAP shares overlapping clinical features with familial adenomatous polyposis (FAP) and Lynch syndrome, making accurate diagnosis challenging. Although cases have been increasingly reported worldwide, PPAP remains underrecognized, particularly in Japan. We present a detailed case of PPAP in a 50‐year‐old woman with a complex cancer history, highlighting the diagnostic challenges and the role of multi‐gene panel testing in establishing the diagnosis.

## 2 Case Report

We report a case of PPAP in a 50‐year‐old woman. PPAP is an autosomal dominant hereditary disorder caused by germline pathogenic variants in the *POLE* or *POLD1* genes, which are responsible for correcting errors during DNA replication. Patients typically develop several dozen colorectal adenomas. Extra‐colonic manifestations have been reported to vary depending on the affected gene: *POLE*‐related PPAP is associated with duodenal adenomas or carcinomas and brain abscesses, while *POLD1*‐related PPAP has been linked to endometrial cancer, breast cancer, and brain tumors [1]. Because PPAP can present with clinical features similar to those of FAP and Lynch syndrome, genetic testing is essential for accurate diagnosis and differentiation.

**FIGURE 1:**
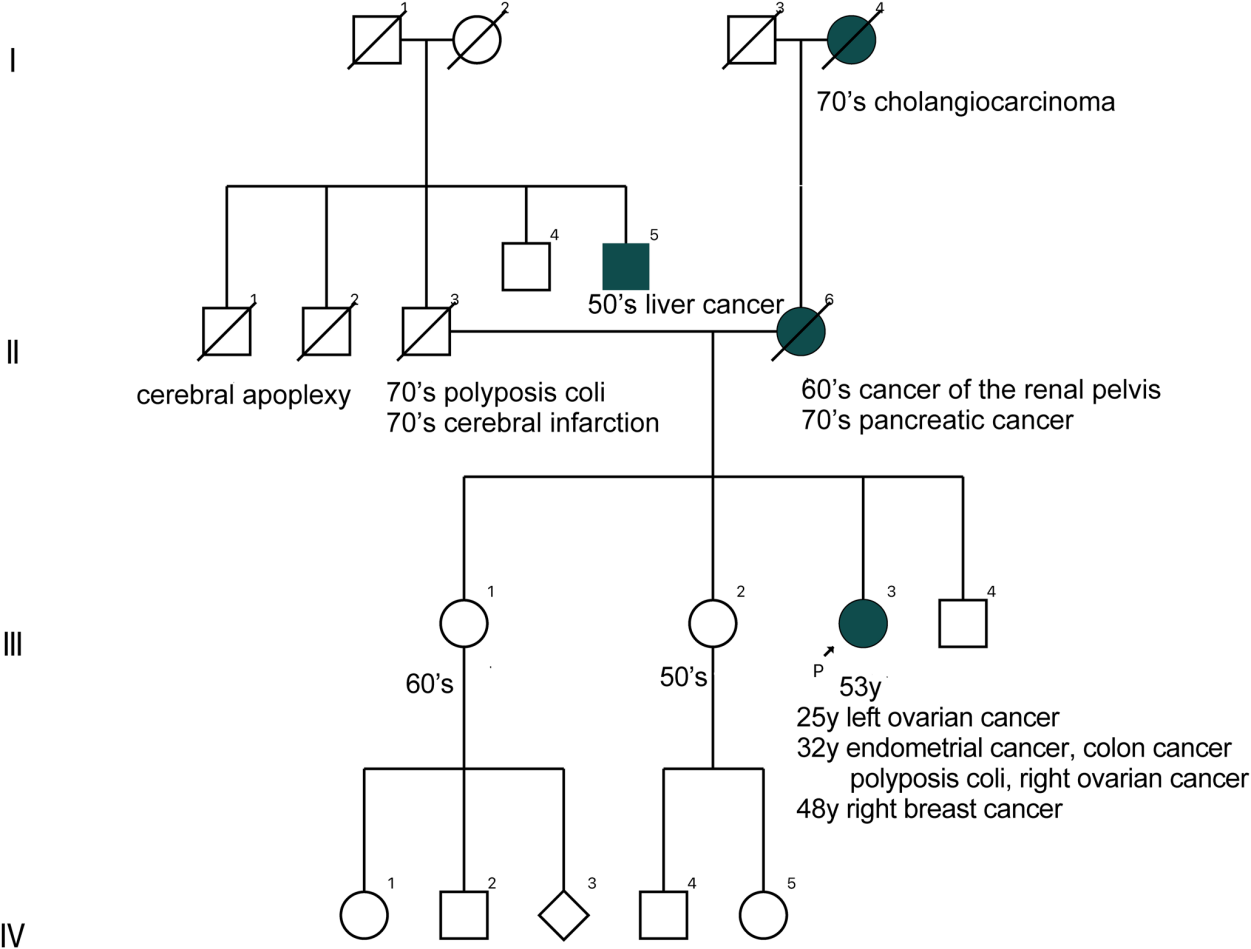
There are no cases of colorectal cancer or gastrointestinal polyposis in patient's family. This case is shown as III‐3.

She had been diagnosed with left ovarian cancer in her 20s and underwent a left adnexectomy. In her 30s, she was diagnosed with endometrial cancer and right ovarian cancer, for which she underwent total hysterectomy, right adnexectomy, omentectomy, and pelvic and para‐aortic lymphadenectomy. Around the same time, early‐stage colon cancer and adenomatous polyposis were also identified, and she underwent total colectomy with ileorectal anastomosis (IRA). Based on the clinical course, FAP of the colon was suspected, and genetic counseling was recommended first. The patient's family history revealed that her mother had pancreatic cancer, and her maternal grandfather had bile duct cancer; however, there was no evidence of colorectal cancer or gastrointestinal polyposis in the family history (Figure 1). In 200X, genetic counseling was conducted at our hospital, and germline pathogenic variants (GPVs) in *APC* and *MUTYH* were analyzed through genetic tests. However, no GPVs were found in *APC*, and a variant of uncertain significance (VUS) was found in *MUTYH*. In 200X + 2, immunohistochemical staining (IHC) of colorectal cancer showed loss of MSH2 and MSH6 proteins; therefore, genetic testing targeting *MSH2* and *MSH6* was performed, but GPVs were not reported. Although the patient's family history raised suspicion for Lynch syndrome, no pathogenic variants were identified in the associated genes, and the diagnosis was therefore considered unlikely. Afterward, the patient developed right breast cancer in her 40s. In 200X + 16, multi‐gene panel testing (MGPT) targeting 22 genes, including *BRCA1/2* and *POLE*, was performed, which revealed a VUS in *POLE* but did not lead to a definite diagnosis. During continued surveillance as a high‐risk case, the interpretation of the variants was revised as additional scientific evidence emerged following the initial analysis, and our institution, which had ordered the test, was subsequently notified.

**FIGURE 2:**
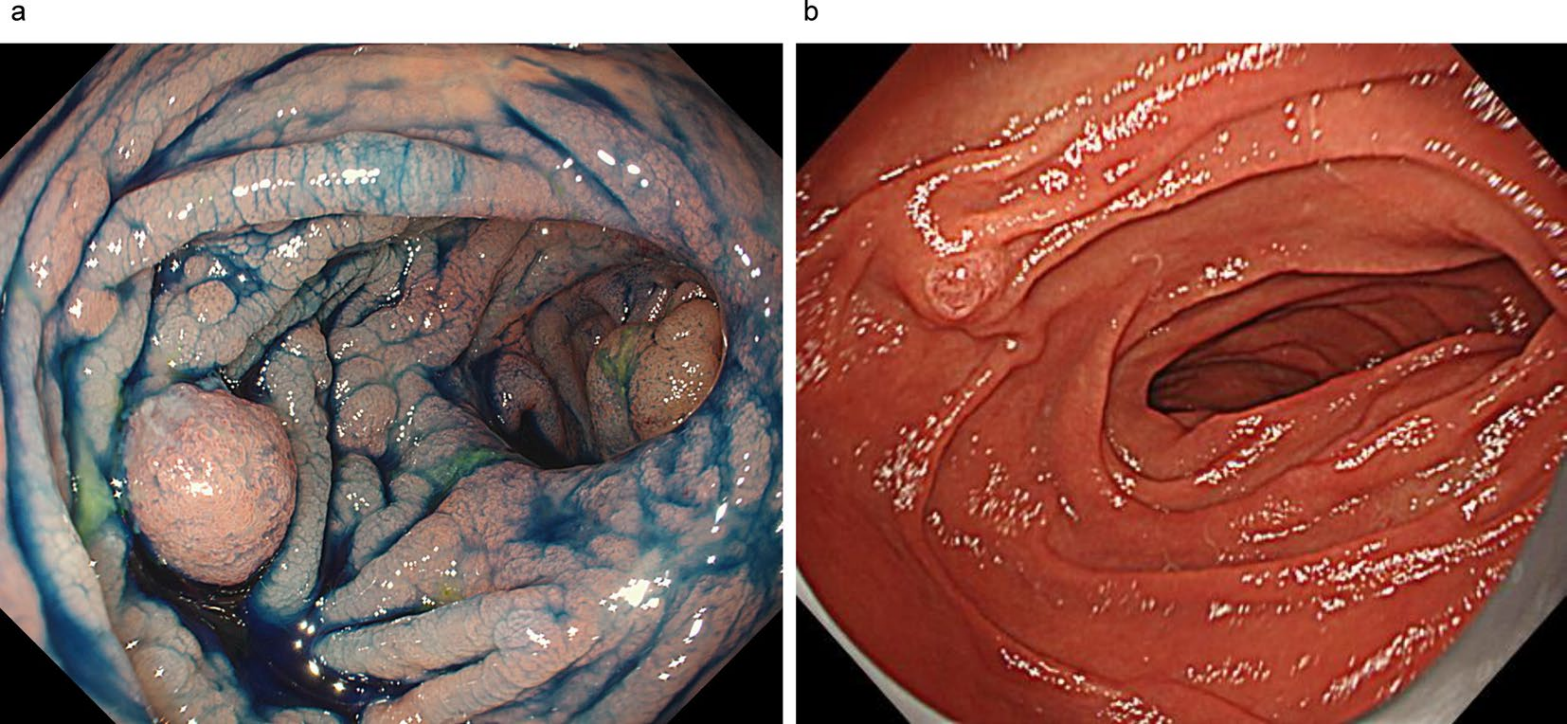
(a) Adenomatous polyposis of the remaining rectum seen on colonoscopy. (b) Descending part of the duodenum. In all cases, a genetic diagnosis was reached through the WGS or MGPT. The patient selected the Colorectal Cancer Panel (VistaSeq), which targets 22 genes associated with colorectal cancers.

In 2020, this variant was reported in a female individual with multiple early‐onset malignancies, including colorectal, ovarian, and endometrial cancer [2]. Moreover, they reported functional evidence from a yeast model indicating a possible impact on protein function. It is presumed that, based on this publication, the testing laboratory reclassified the variant as likely pathogenic. As a result, 5 years after the MGPT, an amended report was issued: VUS in *POLE* was reclassified as likely pathogenic, and the VUS in *MUTYH* was reinterpreted as likely benign. Based on this re‐evaluation, in combination with the patient's clinical phenotype, PPAP's diagnosis was established. The colorectal feature in this patient was the absence of polyps in the early years after IRA, the appearance of solitary polyps that were removed annually in her 30s, and a gradual increase in the number of polyps from her 40s (Figure 2a). In the residual rectum, she developed intramucosal carcinoma twice at 200X + 11 and 200X + 21 years. Furthermore, a tubulovillous adenoma > 30 mm in diameter developed within the J‐pouch at the anastomosis. Regarding upper gastrointestinal diseases, no polyposis of the gastric fundus or duodenal polyps was observed (Figure 2b).

## 3 Discussion

The case reports of PPAP searched in the Central Journal of Medicine and PubMed from 2013 to 2024 revealed four reported cases, including our case, in Japan [3, 4, 5]. (Table 1). Of the three patients other than our patient, one was diagnosed with GPV in *POLE*, and two were diagnosed with GPVs in *POLD1*; all of them had a history of colon cancer. All other patients had a family history of PPAP.

**TABLE 1:** Four patients of polymerase proofreading‐associated polyposis reported in Japan in the past 10 years.

Year/reporter	Age	Sex	Age at initial cancer diagnosis	Age at genetic diagnosis	Initial cancer type	Metachronous cancers	Causative gene	Cancer types among family
Yamaguchi et al. [3]	68	M	68	Unknown	Colon cancer Polyposis coli	Unknown	*POLD1*	Thyroid cancer Gastric cancer Pancreatic cancer
Ito et al. [4]	49	F	49	Unknown	Polyposis coli	Colon cancer Endometrial cancer	*POLD1*	Brain tumor
Miyazaki et al. [5]	43	F	29	Unknown	Colon cancer	Duodenal tumor Endometrial cancer Thyroid cancer	*POLE*	Brain tumor Thyroid cancer
2024 Our case	53	F	25	53	Ovarian cancer	Colon cancer Endometrial cancer Breast cancer	*POLE*	None

Abbreviations: F, female; M, male.

Recommendations for the surveillance of PPAP have not yet been clearly established, as the number of reported cases remains limited, and available evidence is scarce. Consequently, current surveillance strategies are often guided by those used for FAP and Lynch syndrome. Based on the author's clinical perspective, an annual colonoscopy is advisable, with adjustments to the surveillance interval made according to the number and histological characteristics of polyps. For upper gastrointestinal tract evaluation, periodic upper endoscopy every 1–3 years may be appropriate, especially in patients with *POLE* mutations, given reports of duodenal adenomas and carcinomas in this population. For female patients, annual gynecological examinations and transvaginal ultrasonography are recommended, along with endometrial cytology, as needed. Although these strategies are not yet standardized, they represent the author's current approach to the clinical management of PPAP.

MGPT has proven to be a valuable tool for diagnosing adenomatous polyposis, a condition that presents a significant challenge in genetic assessment. This patient underwent prolonged consultations with medical professionals, including genetic counselors, to ascertain a specific phenotype, facilitating subsequent communication and collaboration.

## Ethics Statement

The authors have nothing to report.

## Consent

Informed consent was obtained from this participant.

## Conflicts of Interest

The authors declare no conflicts of interest.

## Data Availability

The data that support the findings of this study are available from the corresponding author upon reasonable request.
